# Screening and validation of potential markers associated with uterine corpus endometrial carcinoma and polycystic ovary syndrome based on bioinformatics methods

**DOI:** 10.3389/fmolb.2023.1192313

**Published:** 2023-06-09

**Authors:** Ruishan Wu, Cailin Wu, Bingming Zhu, Jin Li, Wenzhong Zhao

**Affiliations:** ^1^ NHC Key Laboratory of Male Reproduction and Genetics, Guangdong Provincial Reproductive Science Institute (Guangdong Provincial Fertility Hospital), Guangzhou, China; ^2^ Department of Gynecology, The University of HongKong–Shenzhen Hospital, Shenzhen, China; ^3^ Department of Clinical Laboratory, The First Affiliated Hospital of Jinan University, Guangzhou, China; ^4^ Department of Pain Management, The First Affiliated Hospital of Jinan University, Guangzhou, China

**Keywords:** endometrial carcinoma (UCEC), polycystic ovary syndrome (PCOS), prognostic marker, tumor mutation burden, RTN1

## Abstract

**Background:** Endometrial cancer (UCEC) is a commonly occurring tumor in females, and polycystic ovary syndrome (PCOS) is closely related to UCEC, but the molecular mechanisms remain unclear. This article aims to explore potential molecular mechanisms in UCEC and PCOS, as well as identify prognostic genes for UCEC.

**Methods:** Bioinformatics methods were employed to screen for DEGs in UCEC and PCOS. The shared DEGs were analyzed by constructing a protein-protein interaction (PPI) network using the String database and Cytoscape software. The enrichment analysis was performed using Metascape. The shared DEGs associated with the prognosis of UCEC were identified through univariate and lasso Cox regression methods. A multivariate Cox regression model was constructed and internally validated. The expression and test efficiency of the key prognostic genes were verified using external datasets for UCEC and PCOS. Furthermore, the Gepia database was utilized to analyze the expression of key prognostic genes and their correlation with the disease-free survival (RFS) of UCEC. Tumor mutation burden (TMB), immune infiltration, and the correlation of immune cells were assessed for the prognostic genes of UCEC.

**Results:** There were 151 shared DEGs identified between UCEC and PCOS through bioinformatics screening. These shared DEGs were primarily enriched in leukocyte activation. Following model construction and verification, nine genes were determined to be prognostic for UCEC from the shared DEGs. Among them, TSPYL5, KCNJ15, RTN1, HMOX1, DCAF12L1, VNN2, and ANXA1 were confirmed as prognostic genes in UCEC through external validation. Additionally, RTN1 was identified as a key gene in both UCEC and PCOS. Gepia analysis revealed that higher expression of RTN1 was associated with RFS in UCEC. Immune infiltration analysis of the shared DEGs demonstrated significant differences in the expression of various immune cells between UCEC high and low TMB groups. The seven key prognostic genes in UCEC exhibited regulatory relationships with immune cells.

**Conclusion:** This study identified TSPYL5, KCNJ15, RTN1, HMOX1, DCAF12L1, VNN2, and ANXA1 as the key prognostic DEGs of UCEC. These genes are associated with UCEC survival, TMB, immune cell infiltration, and immune cell regulation. Among them, RTN1 may serve as a potential biomarker for both UCEC and PCOS.

## 1 Introduction

Endometrial cancer (UCEC) is a common malignant tumor in the female reproductive system, specifically in the endometrium. It is more prevalent in developed countries, particularly among middle-aged and menopausal women (Crosbie et al., 2022). The incidence of endometrial cancer is 5.9% (Banz-Jansen et al., 2022) and its mortality rate has been increasing by an average of 1.9% annually (Makker et al., 2021). Obesity is a recognized risk factor for the most common type of UCEC (Rahib et al., 2014; Lauby-Secretan et al., 2016). Polycystic ovary syndrome (PCOS) is a multifactorial, multigene, inflammatory autoimmune disorder that commonly affects reproductive-age women. It is characterized by hyperandrogenism, insulin resistance, ovulation disorders, metabolic disorders, and other features (Liao et al., 2021). PCOS often leads to health problems such as obesity, infertility, metabolic disorders, cardiovascular diseases, and cancers (Patel, 2018; Liao et al., 2021). Obesity and complex metabolic diseases, including PCOS and diabetes, are considered risk factors for UCEC (Lauby-Secretan et al., 2016; Patel, 2018; Liao et al., 2021).

UCEC is pathologically staged into four grades by the International Federation of Gynecology and Obstetrics. A higher grade indicates greater cancer cell proliferation and more challenging treatment. The late-stage survival rate of UCEC is only 17%, highlighting the importance of early diagnosis and treatment in improving survival and prognosis for UCEC patients (Zhang M. et al., 2022). UCEC is commonly treated with surgical resection, hormone therapy, radiotherapy, and chemotherapy. However, in recent years, immunotherapy has emerged as a promising new treatment modality for cancer (Riley et al., 2019; Atsavapranee et al., 2021; El-Mayta et al., 2021). By leveraging checkpoint inhibitors and adoptive cell therapy to manipulate the immune system and enhance its recognition and destruction of cancer cells, cancer immunotherapies offer the potential for durable therapeutic responses in a range of solid tumors and hematologic malignancies (Kennedy and Salama, 2020). Immunotherapy is now being applied to various cancers, including UCEC, providing hope for patients (Makker et al., 2019; Liu et al., 2021b).

High-throughput sequencing can simultaneously detect a large amount of genomic information, with fast and accurate detection speed. This capability greatly enhances people’s ability to assess the risk of complex diseases and the accuracy of targeted drug treatment (Rodriguez and Miller, 2014; Rego and Snyder, 2019). In recent years, high-throughput sequencing has become widely utilized in the medical field. This method enables a profound understanding of disease pathogenesis from a genetic perspective. (Rego and Snyder, 2019). With the advancement and application of high-throughput sequencing technology, the diagnosis and treatment of UCEC have shifted from histology to genomics. Genomic evidence, along with clinicopathological criteria, provides crucial information for UCEC treatment and enables the delivery of improved quality and personalized services to patients (Mitric and Bernardini, 2022). Bioinformatics, combined with high-throughput sequencing, can efficiently screen and verify key disease-related genes. Moreover, it can predict disease risk.

While PCOS has been linked to an increased risk of UCEC (Lauby-Secretan et al., 2016; Patel, 2018; Liao et al., 2021), meta-analyses have shown a significant association between PCOS and the pathogenesis of UCEC (Barry et al., 2014; Li Z. et al., 2022). However, the molecular mechanisms underlying this link remain unclear. Understanding the pathophysiological connection between UCEC and PCOS is crucial for developing effective clinical treatment strategies. To address this, we employed bioinformatics methods to screen for shared DEGs between UCEC and PCOS. We visualized the protein-protein interaction (PPI) network of these genes using the String database and Cytoscape software. Next, we utilized lasso Cox regression, and univariate and multivariate Cox regression methods to identify key shared differentially expressed genes (DEGs) associated with the prognosis of UCEC. We then constructed and validated the model. Using the Gepia database, we analyzed the expression levels and RFS of key shared DEGs across different pathological stages of UCEC. Finally, we conducted tumor mutation burden (TMB) and immune infiltration analysis on both the shared and key prognostic genes for UCEC and PCOS.

## 2 Materials and methods

### 2.1 Data download

The data for UCEC were obtained by searching the keywords “transcriptome profiling, Gene Expression Quantification, TCGA-UCEC” from The Cancer Genome Atlas (TCGA) database (https://portal.gdc.cancer.gov/). Keywords such as “polycystic ovary syndrome, PCOS” were searched in the Gene Expression Omnibus (GEO) database to retrieve the gene expression profile of PCOS. Additionally, keywords such as “Endometrial cancer, EC, UCEC” were searched in the GEO database to obtain the gene expression profile of UCEC. The inclusion criteria for the PCOS and UCEC datasets were as follows: 1) Gene expression profiles must include both a case group and a control group. 2) Datasets should have available raw data or analyzable data.

### 2.2 Screening for DEGs

The UCEC data matrix file was downloaded from the TCGA database, the PCOS data matrix file was downloaded from the GEO database, and the “limma” R package, a microarray linear model, was utilized to correct the data and analyze the fold change (FC). The log2 FC of each gene was used to determine its rank in the final gene list. Genes with a *p* < 0.05 and |log2 FC| ≥ 0.5 were considered significant DEGs and were subjected to further analysis.

### 2.3 Identifying shared DEGs for UCEC and PCOS

A Venn diagram was constructed using the online website (http://www.ehbio.com/test/venn/#/) to identify the intersection of DEGs between UCEC and PCOS, representing the shared DEGs for both conditions.

### 2.4 Construction and enrichment analysis of shared DEGs’ PPI network

“String” is an online network tool that can predict protein association between proteins and draw a PPI network (von Mering et al., 2003; Szklarczyk et al., 2017; Szklarczyk et al., 2023). In this study, we utilized String to analyze the protein interaction network of shared DEGs between UCEC and PCOS. The String using method is as follows: Log in to the String database (https://string-db.org/), click “Multiple Proteins”, enter shared DEGs into “List of Names”, select “*Homo sapiens*” for organisms, and click “search” to perform protein mutual assistance on shared DEGs network analysis, when greater than 0.4 indicates statistical significance.

Cytoscape software, when combined with extensive databases of PPI, protein-DNA, and genetic interactions, is a powerful tool (Shannon et al., 2003). In this study, the String database and Cytoscape software were employed to map and visualize the PPI networks of shared DEGs between UCEC and PCOS. The CytoNCA plug-in in Cytoscape software (version 3.9.1) was utilized to perform topological analysis and betweenness centrality (BC) screening of the resulting protein interaction network data (Tang et al., 2015). Subsequently, the shared DEGs were ranked based on their degree values. A PPI network map representing the shared DEGs between UCEC and PCOS was generated, and the top 10 shared DEGs with the highest degree values were identified and visualized.

Metascape (https://metascape.org/gp/index.html#/main/step1) is an online platform that provides comprehensive annotation and analysis resources for gene lists (Zhou et al., 2019). In this study, we utilized Metascape to perform gene ontology (GO) and Kyoto Encyclopedia of Genes and Genomes (KEGG) pathway enrichment analyses on the shared DEGs between UCEC and PCOS.

### 2.5 Establishment of the UCEC prognostic gene model

The UCEC prognostic model was developed using shared DEGs data from UCEC and PCOS. Univariate Cox analysis was employed to identify UCEC prognostic genes. Lasso analysis and cross-validation were used to address the issue of overfitting by eliminating highly correlated genes. Subsequently, multivariate Cox regression was performed to further reduce dimensionality. Genes associated with UCEC survival in multivariate Cox regression were extracted as prognostic genes. These prognostic genes were utilized to construct the model and generate forest plots.

### 2.6 Internal validation of the prognostic model on the TCGA database

The TCGA-UCEC data were divided into training (50%) and testing datasets (50%) for model internal validation. The obtained model was then used to predict the risk of training and testing samples. Based on the median value of the training dataset, patients in both datasets were divided into high-risk and low-risk groups. The risk value was calculated using the formula: Risk value = expression level of gene 1 * coef1 + expression level of gene 2 * coef2 +. expression level of gene n * coefn. To compare the difference in overall survival between the high-risk and low-risk groups, Kaplan-Meier (KM) analysis from the “survival” package was performed on the training and testing datasets. Additionally, the predictive performance of the model was evaluated using receiver operating characteristic (ROC) curves generated for 1, 3, and 5 years using the “survivalROC” package. These ROC curves were created for both the training and testing datasets. Risk values for the training and testing datasets were obtained and used to generate risk score distribution maps, survival distribution maps, and expression heat maps to visualize the results.

### 2.7 External validation of prognostic genes, and the establishment of key genes

External validation datasets for UCEC and PCOS prognostic genes were obtained from the GEO database, meeting the selection criteria. The expressions of key shared DEGs from UCEC and PCOS were verified using the external validation datasets. UCEC prognostic key genes were selected based on significant differences between normal controls and UCEC in the external validation dataset. Similarly, PCOS key genes were chosen by identifying the key shared DEGs that displayed significant differences between normal controls and PCOS in the external validation dataset. The intersection of externally validated key genes for both UCEC and PCOS represents potential markers for both diseases. The predictive performance of the genes was assessed using ROC curves generated with the “qROC” package. A ROC curve greater than 0.6 was considered indicative of good predictive performance for the gene.

### 2.8 Expression and survival analysis by Gepia

The Gepia database (http://gepia.cancer-pku.cn/) is a valuable resource for data mining and gaining insights into gene function (Tang et al., 2017). In this study, we utilized the “survival” module of the Gepia database to explore the association between the expression of key prognostic genes and the RFS in UCEC.

### 2.9 Immune infiltration analysis of shared DEGs

Cibersort immune cell infiltration analysis was conducted on the shared DEGs using the “limma” R package. A bar plot distribution of the shared DEGs was generated for 22 immune cell subsets. The shared genes were then categorized into high and low TMB groups in UCEC, and the differences in immune cell infiltrate between these groups were compared.

### 2.10 Immune correlates of key prognostic genes

To examine the correlation between key prognostic genes in UCEC and immune cells, we utilized the R packages “reshape2”, “ggpubr”, and “ggExtra”. Subsequently, we employed R software to create a lollipop diagram to visualize the correlation between the key prognostic genes and immune cells.

## 3 Results

### 3.1 Identification of DEGs

**FIGURE 1:**
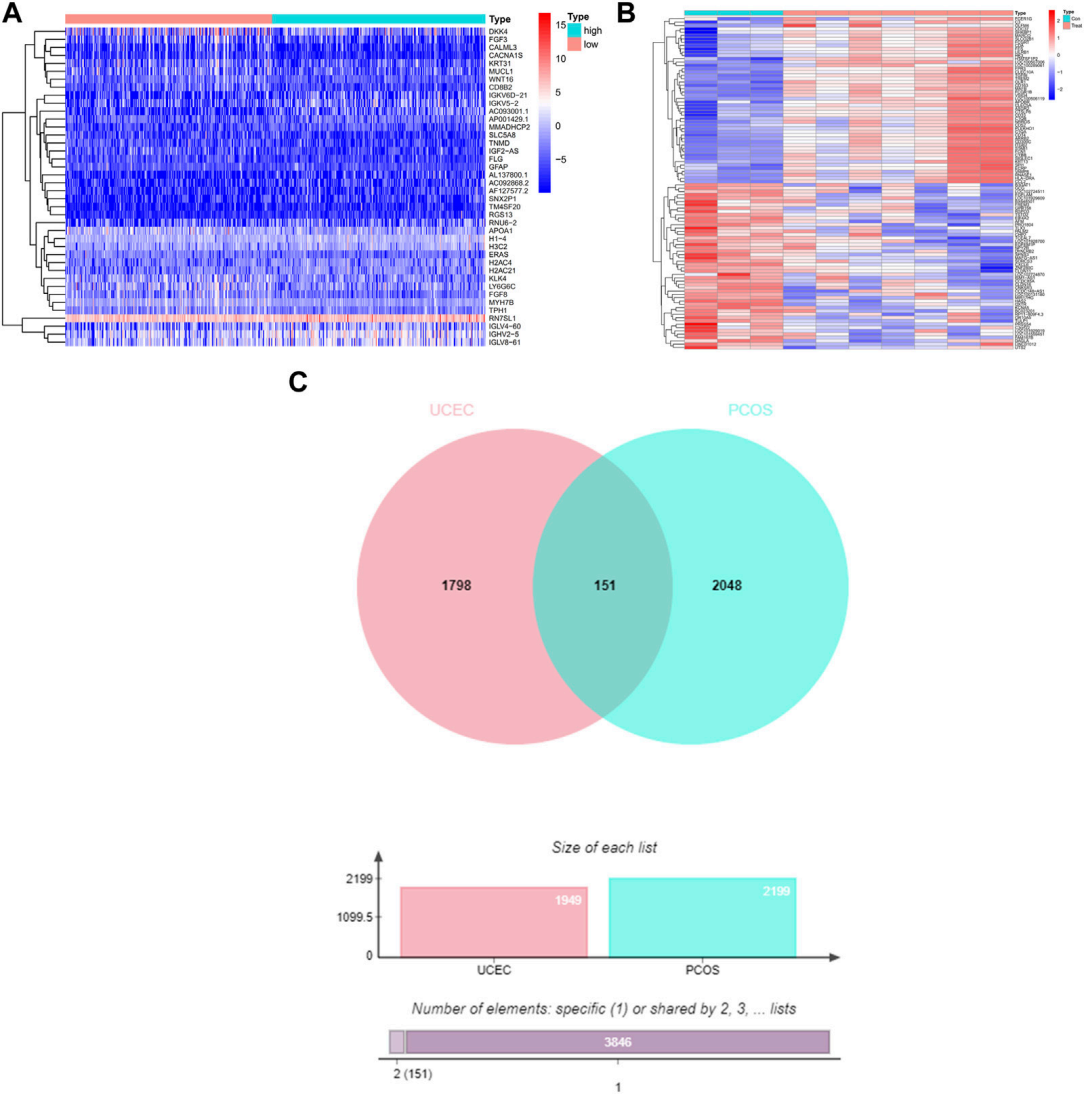
The DEGs and shared DEGs for UCEC and PCOS. **(A)** The heat map represents the DEGs of UCEC, with a total of 1949 UCEC DEGs. **(B)** The heat map represents the DEGs of PCOS, with a total of 2199 PCOS DEGs. **(C)** The Venn diagram illustrates the shared DEGs between UCEC and PCOS, revealing 151 shared genes in both UCEC and PCOS.

The TCGA-UCEC database consists of 35 cases of normal endometrium and 554 cases of UCEC. Through screening, a total of 1949 UCEC DEGs were identified, including 958 upregulated differential genes and 991 downregulated differential genes. The UCEC DEGs are presented in the heat map (Figure 1A). The analysis of PCOS dataset GSE34526 from the GEO database was performed using the Affymetrix Human Genome U133 Plus 2.0 Array platform, resulting in the identification of 2199 DEGs meeting the conditions, including 1373 upregulated differential genes and 826 downregulated differential genes. The PCOS DEGs are shown in the heat map (Figure 1B).

### 3.2 Confirming the shared DEGs for UCEC and PCOS

The Venn diagram demonstrates the presence of 151 shared DEGs between UCEC and PCOS (Figure 1C). Among the 151 shared differential genes, 73 were upregulated and 78 were downregulated in UCEC (Supplementary Figure S1).

### 3.3 PPI network and enrichment analysis of shared DEGs for UCEC and PCOS

**FIGURE 2:**
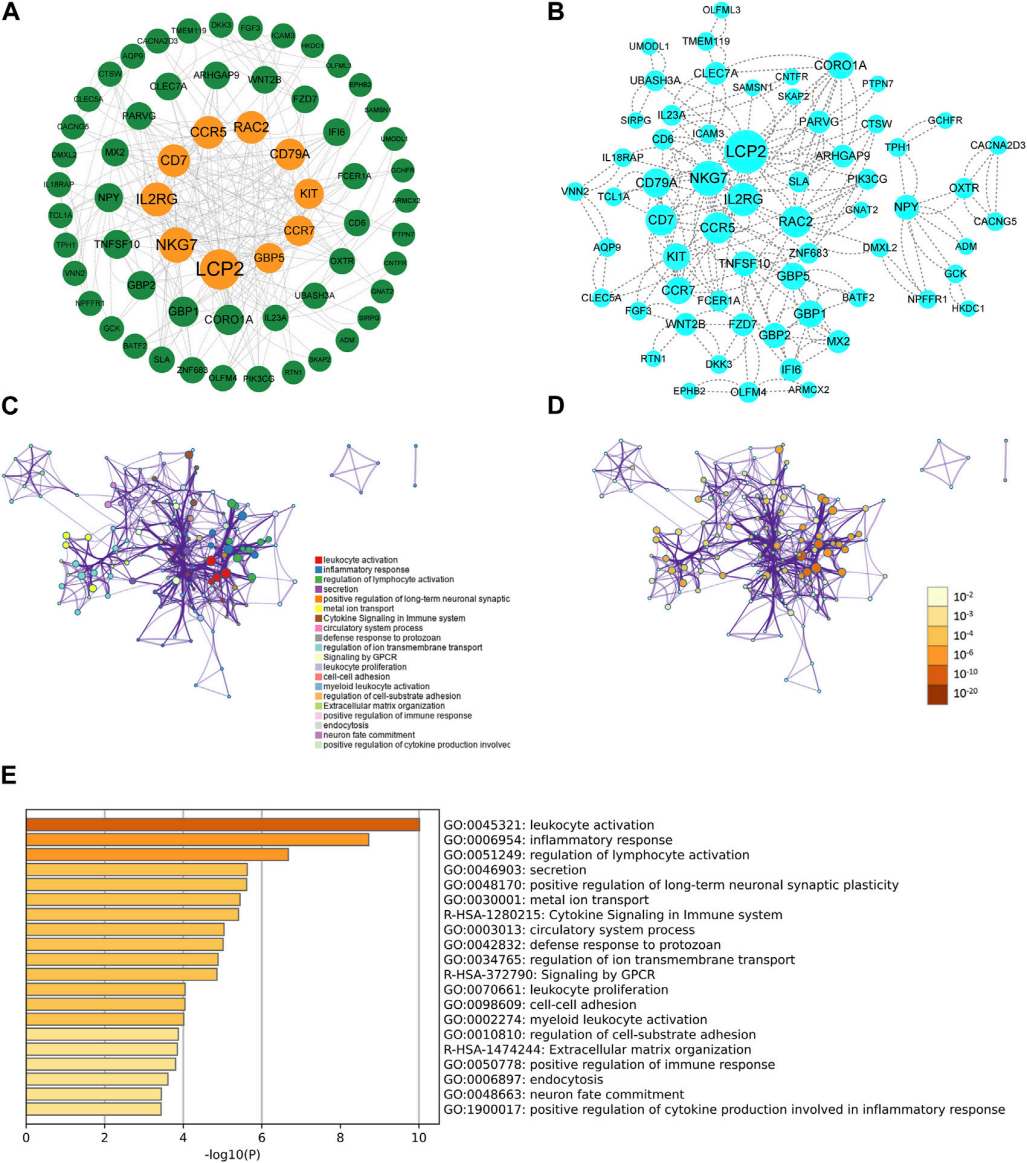
The PPI network and enrichment analysis of shared DEGs for UCEC and PCOS. **(A)** The key shared DEGs of UCEC and PCOS. Among the shared DEGs, LCP2, NKG7, IL2RG, CD7, CCR5, RAC2, CD79A, KIT, CCR7, and GBP5 were the top ten genes based on degree. **(B)** The PPI network of shared DEGs for UCEC and PCOS. The shared DEGs enrichment GO color by cluster **(C)** and *p*-value **(D)**. **(E)** The bar chart of enrichment analysis of shared DEGs. The result showed that they mainly enrich leukocyte activation, inflammatory response, and regulation of lymphocyte activation.

Based on the interactions of shared DEGs obtained from the Sting database, Cytoscape software was used to visualize the key shared DEGs (Figure 2A) and the PPI network diagram of shared genes (Figure 2B). It was determined that the top ten genes based on degree were LCP2, NKG7, IL2RG, CD7, CCR5, RAC2, CD79A, KIT, CCR7, and GBP5(Table 1; Figures 2A, B). The shared DEGs were enriched using the Metascape online database, and PPI networks were based on cluster analysis (Figure 2C) and *p*-value (Figure 2D). The top three enrichment analyses were leukocyte activation, inflammatory response, and regulation of lymphocyte activation as the most significant processes (Figure 2E).

**TABLE 1:** The top ten degrees of shared DEGs in PCOS and UCEC.

Name	Betweenness	Closeness	Degree	Shared name
LCP2	1247.608	0.371951	28	LCP2
NKG7	438.321	0.308081	20	NKG7
IL2RG	843.6401	0.365269	18	IL2RG
CD7	157.3024	0.309645	16	CD7
CCR5	1065.951	0.335165	16	CCR5
RAC2	1717.966	0.363095	16	RAC2
CD79A	126.3662	0.306533	14	CD79A
KIT	168.2864	0.283721	12	KIT
CCR7	59.69935	0.291866	12	CCR7
GBP5	333.5	0.262931	12	GBP5

### 3.4 Establishment of shared DEGs prediction model for UCEC

**FIGURE 3:**
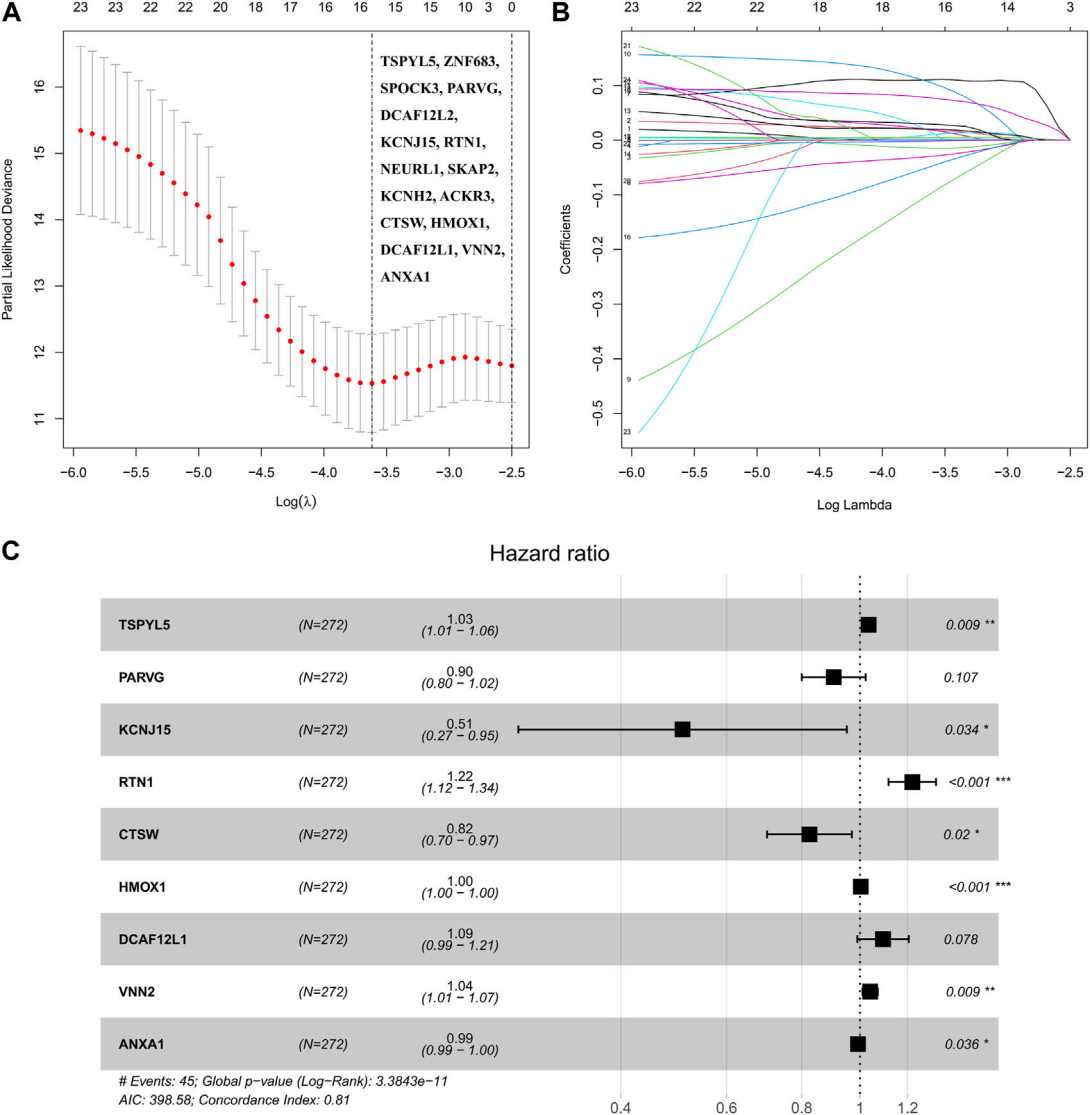
The robust prognostic shared DEGs of UCEC and PCOS were based on the Cox regression model. **(A)** The partial likelihood bias and log *λ* of lasso Coss analysis, and Lasso regression identified 16 candidate genes. **(B)** The coefficient and log *λ* of lasso Coss analysis. **(C)** The forest plot of nine prognostic shared DEGs was based on the multivariate Cox regression model.

The analysis of the 151 shared DEGs between UCEC and PCOS involved performing univariate Cox regression, which revealed 23 DEGs associated with UCEC survival. The lasso regression lambda optimal algorithm was employed, and tenfold cross-validation identified an optimal lambda value of −3.6 (Figure 3A). Utilizing lasso regression, 16 candidate genes associated with UCEC prognosis were identified, each having non-zero lasso coefficients (Figure 3B). Those genes include TSPYL5, ZNF683, SPOCK3, PARVG, DCAF12L2, KCNJ15, RTN1, NEURL1, SKAP2, KCNH2, ACKR3, CTSW, HMOX1, DCAF12L1, VNN2, and ANXA1. Subsequently, a multivariate Cox regression model was constructed, resulting in the identification of nine prognostic markers for UCEC. These markes are TSPYL5, PARVG, KCNJ15, RTN1, CTSW, HMOX1, DCAF12L1, VNN2, and ANXA1 (Table 2; Figure 3C). Among them, VNN2, PARVG, CTSW, HMOX1, and ANXA1 are upregulated in UCEC, while DCAF12L1, KCNJ15, TSPYL5, and RTN1 are downregulated in UCEC (Supplementary Figure S2).

**TABLE 2:** Multivariate Cox regression analyses of nine shared DEGs.

ID	Coef	HR	HR.95L	HR.95H	*p*-value
TSPYL5	0.033701	1.034275	1.008409	1.060806	0.009109	
PARVG	−0.10052	0.904369	0.800222	1.022071	0.107344	
KCNJ15	−0.67959	0.506825	0.27009	0.951057	0.034326	
RTN1	0.200334	1.221811	1.115558	1.338183	1.59E-05	
CTSW	−0.19341	0.824144	0.700535	0.969563	0.01966	
HMOX1	0.002834	1.002838	1.001585	1.004093	8.97E-06	
DCAF12L1	0.088495	1.092529	0.990085	1.205574	0.078136	
VNN2	0.039031	1.039803	1.0096	1.07091	0.009454	
ANXA1	−0.00733	0.992698	0.985906	0.999537	0.03643	

### 3.5 Internal validation of shared DEGs prediction models

**FIGURE 4:**
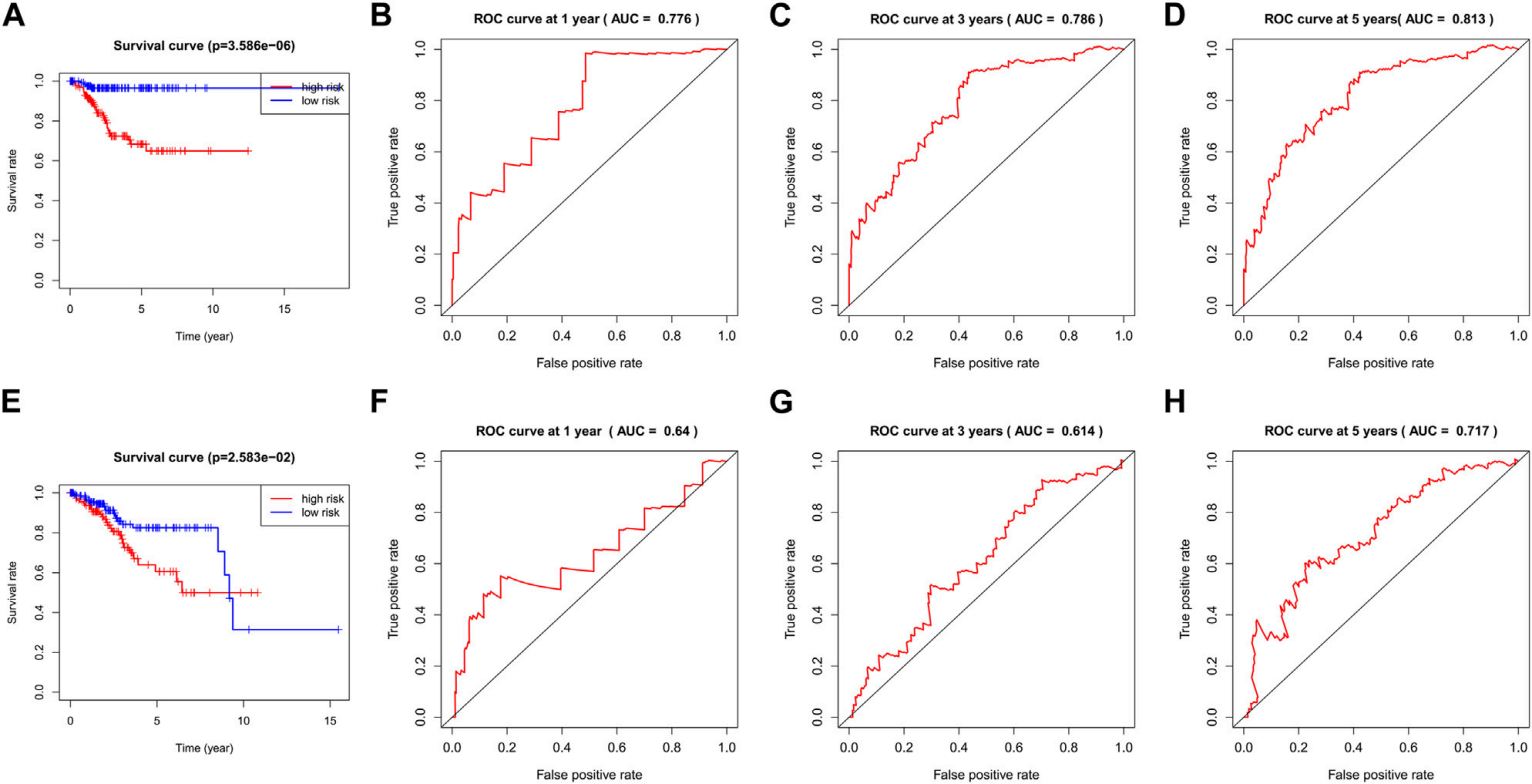
The KM survival curve and ROC curve in the training and testing dataset. The survival curve in the high- and low-risk groups of the training dataset **(A)** and testing dataset **(E)** indicated that the high-risk group had a shorter survival time compared to the low-risk group (*p*< 0.05). The ROC curve values at 1 **(B)**, 3 **(C)**, and 5 **(D)** years in the training dataset were 0.776, 0.786, and 0.813 (AUC>0.6). The ROC curve values at 1 **(F)**, 3 **(G)**, and 5 **(H)** years in the testing dataset were 0.64, 0.614, and 0.717 (AUC>0.6).

The TCGA-UCEC data were divided into a training dataset (272 cases) and a testing dataset (272 cases). The median risk value of the training dataset was determined to be 1.653732. Using this median risk value as the cutoff, both the training datasets and testing datasets were divided into two groups: high-risk and low-risk. Each group consisted of 136 UCEC cases. The risk value proved to be a reliable predictor. The KM survival curves for the high-risk and low-risk groups in both the training and testing datasets indicated that the high-risk group had a shorter survival time compared to the low-risk group (*p*< 0.05, Figures 4A, E).

The ROC curve values at 1, 3, and 5 years in the training dataset were 0.776, 0.786, and 0.813, respectively, (Figures 4B–D). The testing dataset was used to validate the model and the corresponding ROC curve values at 1, 3, and 5 years in the testing dataset were 0.64, 0.614, and 0.717, respectively, (Figures 4F–H). These ROC curve values indicate that the model constructed by the nine prognostic genes in both the UCEC training dataset and the testing dataset performed well, as all values were greater than 0.6.

**FIGURE 5:**
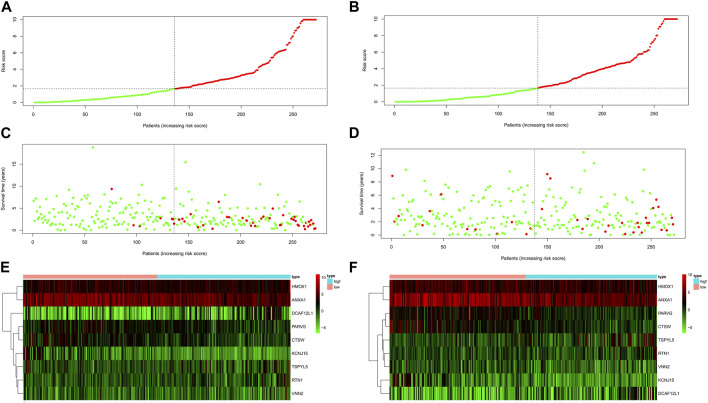
The risk score of the nine shared DEGs in UCEC. The risk score distribution of patients in the training dataset **(A)** and testing dataset **(B)**. UCEC patient survival in the training dataset **(C)** and testing dataset **(D)**. The higher risk scores were associated with increased mortality rates in the training and testing dataset. The expression profiles of nine shared DEGs in the training dataset **(E)** and testing dataset **(F)**.

The survival scores and status of the UCEC training dataset indicated that higher risk scores were associated with increased mortality rates (Figures 5A, C). Furthermore, when validating the risk score of the model using the testing dataset, the survival scores and status of the high-risk and low-risk groups also demonstrated that higher-risk scores were correlated with higher mortality rates (Figures 5B, D). The expression levels of the nine key shared DEGs in both the training and testing datasets were visualized using a heat map (Figures 5E, F).

### 3.6 External validation of prognostic genes

**FIGURE 6:**
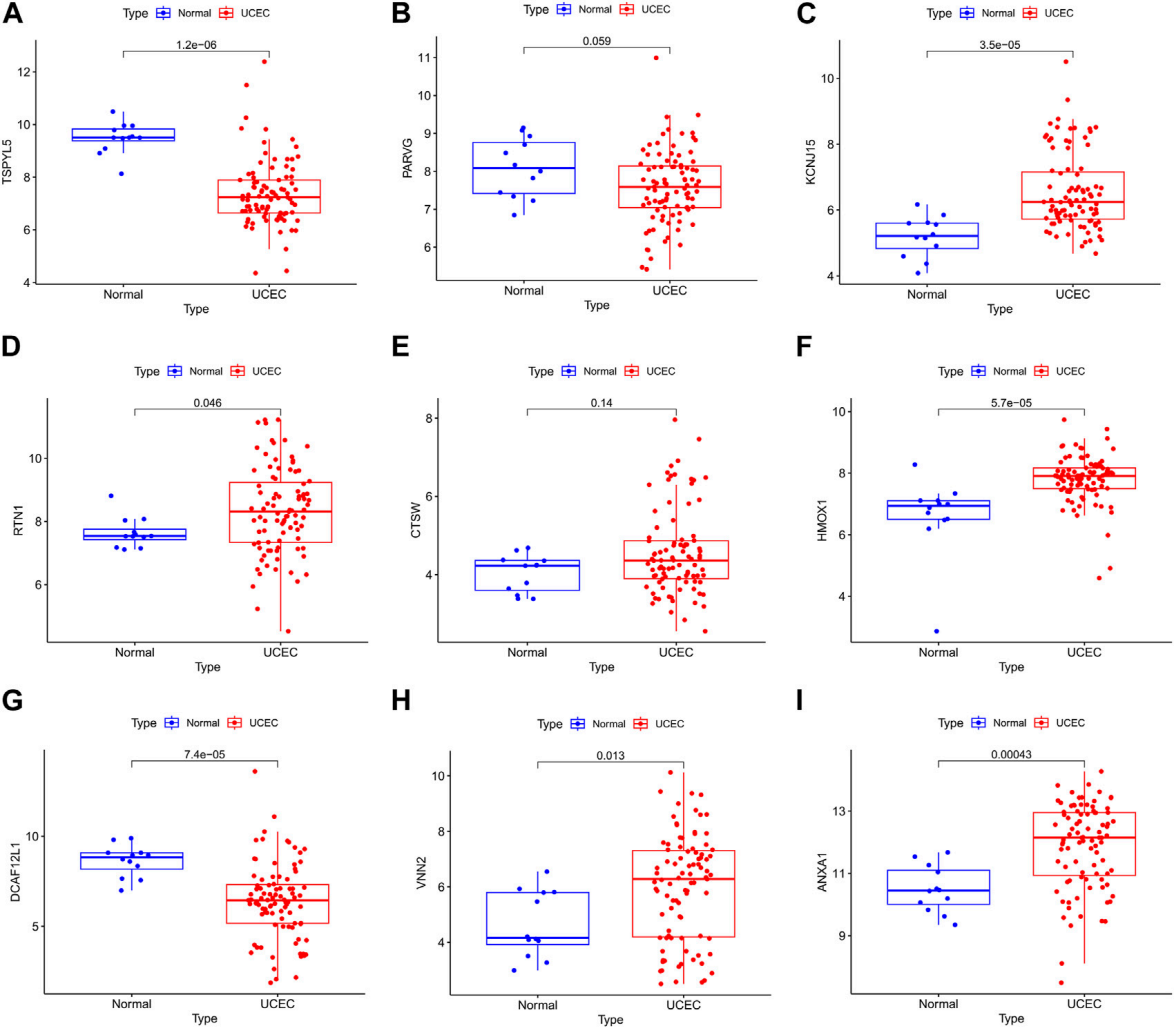
The bar chart of the nine DEGs in UCEC external verification. The expressions of TSPYL5 **(A)**, KCNJ15 **(C)**, RTN1 **(D)**, HMOX1 **(F)**, DCAF12L1 **(G)**, VNN2 **(H)**, and ANXA1 **(I)** were significantly different in normal control and UCEC (*p*> 0.05). The expressions of PARVG **(B)** and CTSW **(E)** were not different in normal control and UCEC (*p*> 0.05).

**FIGURE 7:**
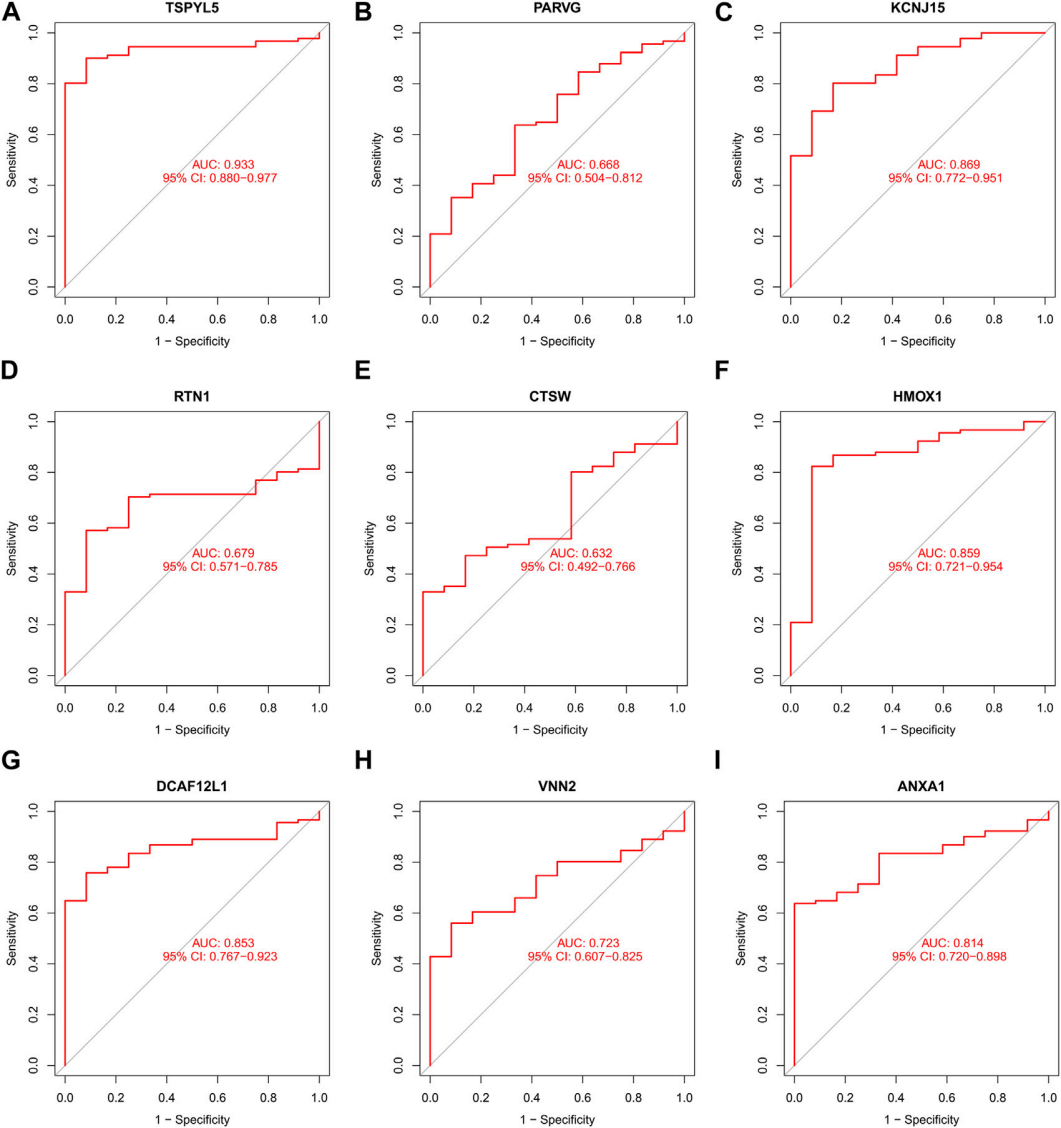
The ROC curve of the nine DEGs in UCEC external verification. The ROC curves of those nine shared DEGs had good predictive performances. The AUCs of TSPYL5 **(A)**, PARVG **(B)**, KCNJ15 **(C)**, RTN1 **(D)**, CTSW **(E)**, HMOX1 **(F)**, DCAF12L1 **(G)**, VNN2 **(H)**, and ANXA1 **(I)** were 0.933, 0.668, 0.869, 0.679, 0.632, 0.859, 0.853, 0.723, and 0.814, respectively.

**FIGURE 8:**
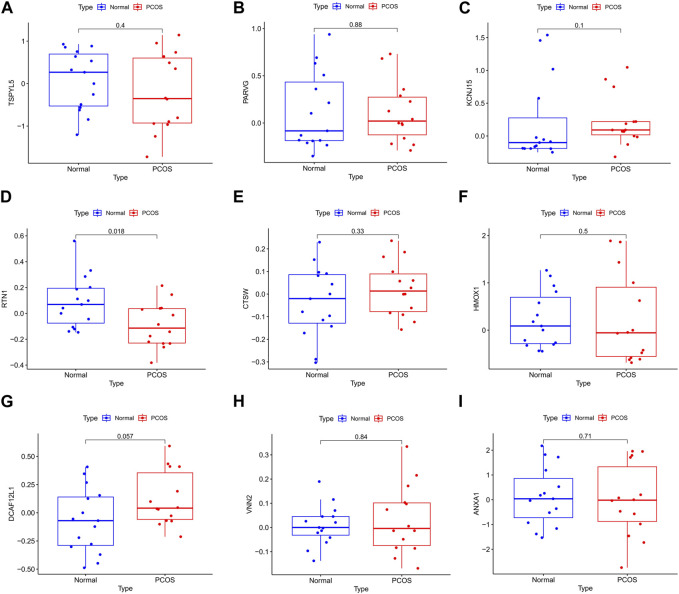
The bar chart of the nine DEGs in PCOS external verification. The expression of RTN1 **(D)** was significantly different in normal control and PCOS (*p*< 0.05). The expressions of TSPYL5 **(A)**, PARVG **(B),** KCNJ15 **(C)**, CTSW **(E)**, HMOX1 **(F)**, DCAF12L1 **(G)**, VNN2 **(H)**, and ANXA1 **(I)** were not different in normal control and PCOS (*p*> 0.05).

**FIGURE 9:**
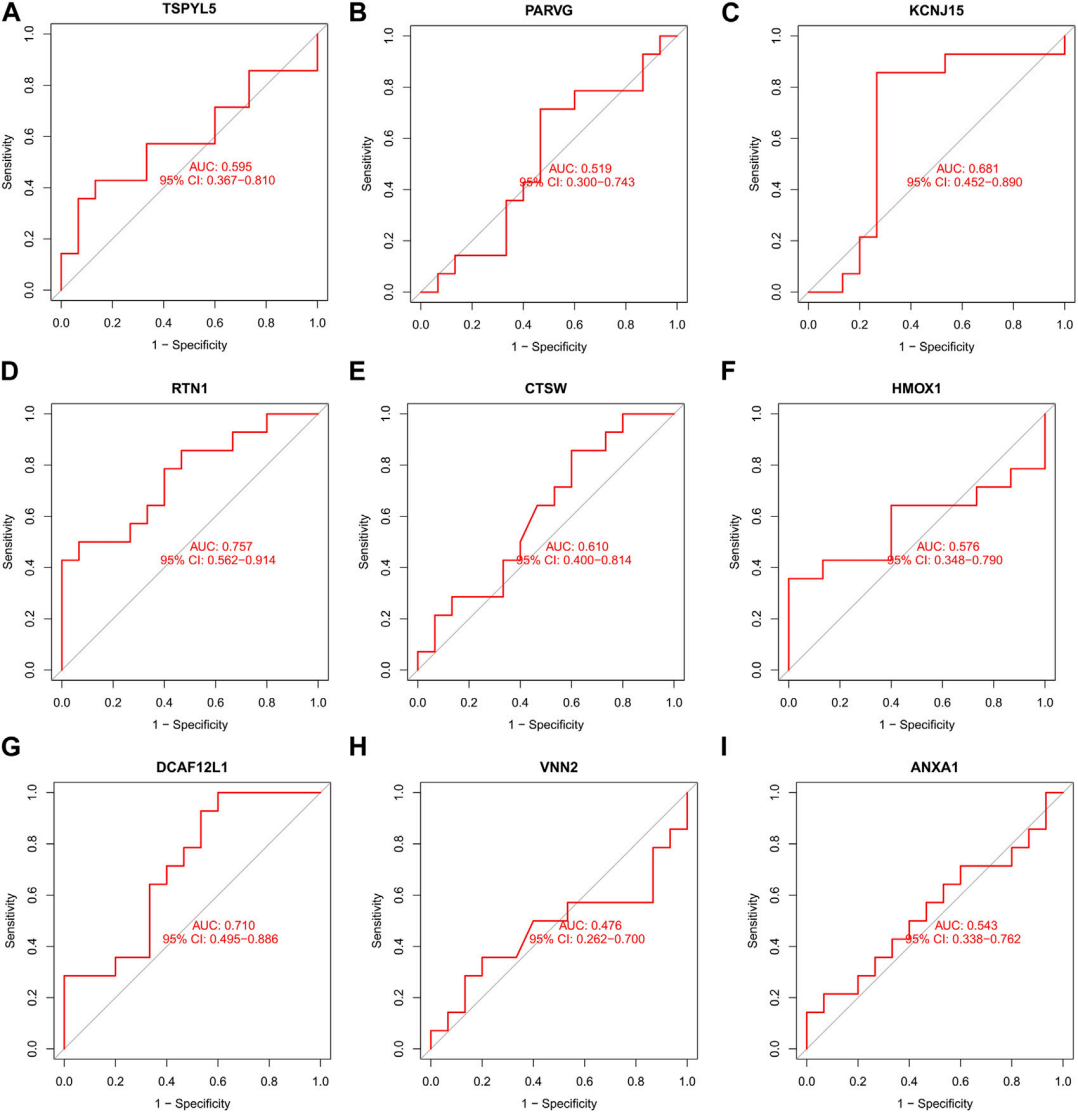
The ROC curve of the nine DEGs in PCOS external verification. The ROC curves of KCNJ15 **(C)**, RTN1 **(D)**, CTSW **(E)**, and DCAF12L1 **(G)** were 0.681, 0.757, 0.610, and 0.710, respectively, indicating good predictive performance (AUC>0.6). The ROC curves of TSPYL5 **(A)**, PARVG **(B),** KCNJ15 **(C)**, HMOX1 **(F)**, VNN2 **(H)**, and ANXA1 **(I)** had no predictive performances (AUC<0.6).

The UCEC dataset GSE17025 was used as an external validation dataset to validate the prognostic genes TSPYL5, PARVG, KCNJ15, RTN1, CTSW, HMOX1, DCAF12L1, VNN2, and ANXA1. Among them, the expressions of TSPYL5, KCNJ15, RTN1, HMOX1, DCAF12L1, VNN2, and ANXA1 exhibited significant differences between normal controls and UCEC (*p*< 0.05, Figure 6), confirming their status as key prognostic genes for UCEC. The ROC curves for all nine prognostic genes in the UCEC external validation set were over 0.6 (Figure 7A–I), indicating their good predictive performance. The PCOS dataset GSE48301 was used as an external validation dataset to validate the same nine prognostic genes. The results showed that RTN1 exhibited a significant difference between normal controls and PCOS (*p* < 0.05, Figure 8D). However, the expressions of TSPYL5 (Figure 8A), PARVG (Figure 8B), KCNJ15 (Figure 8C), CTSW (Figure 8E), HMOX1 (Figure 8F), DCAF12L1 (Figure 8G), VNN2 (Figure 8H), and ANXA1 (Figure 8I) were not different in normal control and PCOS (p > 0.05). Moreover, the ROC curves for KCNJ15 (Figure 9C), RTN1 (Figure 9D), CTSW (Figure 9E), and DCAF12L1 (Figure 9G) demonstrated good predictive performance (AUC>0.6). Therefore, RTN1 was identified as a potential marker in both UCEC and PCOS.

### 3.7 RFS and expression analysis by Gepia

**FIGURE 10:**
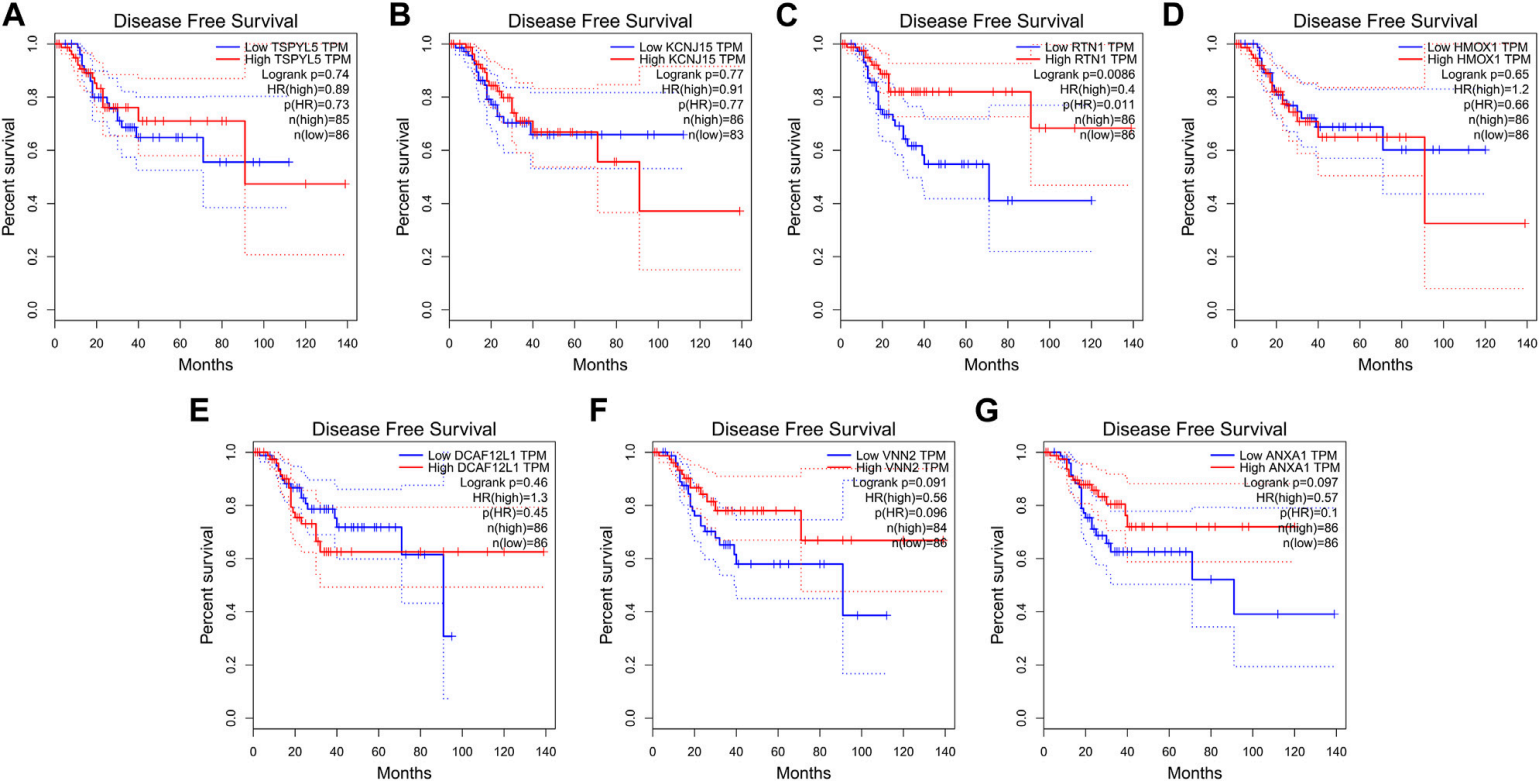
RFS and expression of the seven key prognostic genes in UCEC. The expression of RTN1 **(C)** was related to the RFS of UCEC (*p*< 0.05). The expressions of TSPYL5 **(A)**, KCNJ15 **(B)**, HMOX1 **(D)**, DCAF12L1 **(E)**, VNN2 **(F)**, and ANXA1 **(G)** were not related to RFS of UCEC (*p*> 0.05).

The expressions of the key prognostic genes TSPYL5, KCNJ15, RTN1, HMOX1, DCAF12L1, VNN2, and ANXA1 in UCEC were analyzed using Gepia. The results showed that higher expression of RTN1 was associated with longer RFS in UCEC patients (*p*< 0.05, Figure 10C). However, the expressions of TSPYL5 (Figure 10A), KCNJ15 (Figure 10B), HMOX1 (Figure 10D), DCAF12L1 (Figure 10E), VNN2 (Figure 10F), and ANXA1 (Figure 10G) were not found to be related to the RFS of UCEC patients (*p*> 0.05).

### 3.8 Immune infiltration analysis of shared DEGs

**FIGURE 11:**
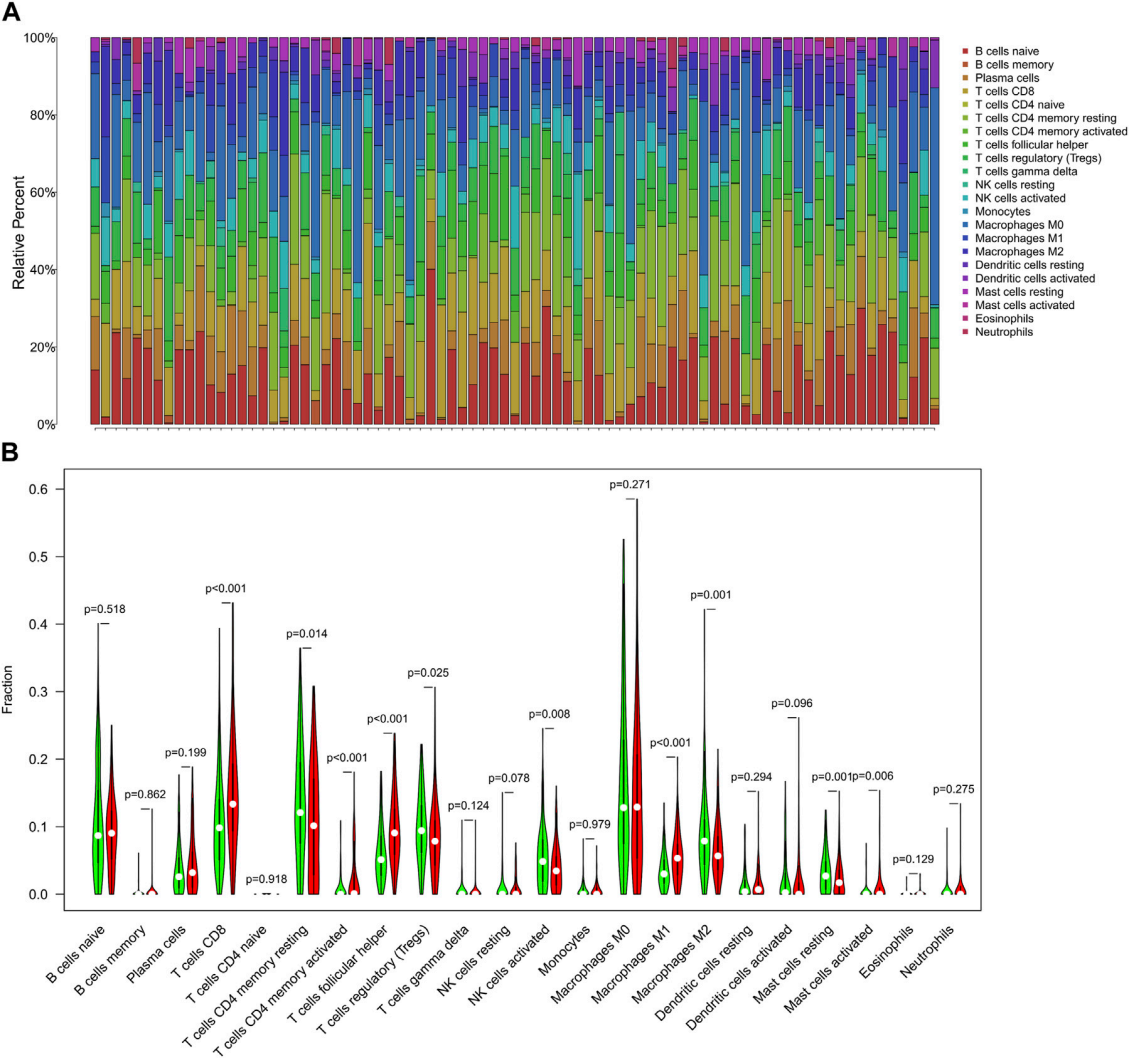
The immune infiltration analysis of shared DEGs. **(A)** The bar chart of shared DEGs and immune cell subsets. Each column represents a sample, and different colors represent different immune cells. **(B)** The violin diagram of the TMB in shared DEGs. The Wilcoxon rank-sum test showed that T cells CD8 (*p*< 0.001), T cells CD4 memory resting (*p* = 0.014), T cells CD4 memory activated (*p*< 0.001), T cells follicular helper (*p* < 0.001), T cells regulatory (*p* = 0.025), NK cells activated (*p* = 0.008), Macrophages M1 (*p* < 0.001), Macrophages M2 (*p* = 0.001), Mast cells resting (*p* = 0.001), and Mast cells activated (*p* = 0.006) had a difference in high and low TMB groups.

Based on the Cibersort algorithm, we analyzed the infiltration of immune cells in UCEC and PCOS using the shared DEGs. Figure 11A shows the proportion of 22 immune cell subsets in the 151 shared DEGs. We processed the shared DEGs using R software and stratified them into high and low TMB groups. The results indicated that T cells CD8, T cells CD4 memory resting, T cells CD4 memory activated, T cells follicular helper, T cells regulatory, NK cells activated, macrophages M1, macrophages M2, mast cells resting, and mast cells activated exhibited significantly different expressions between the UCEC high and low TMB groups (*p* < 0.05, Figure 11B).

### 3.9 Regulatory relationship between key prognostic genes and immune cells in UCEC

**FIGURE 12:**
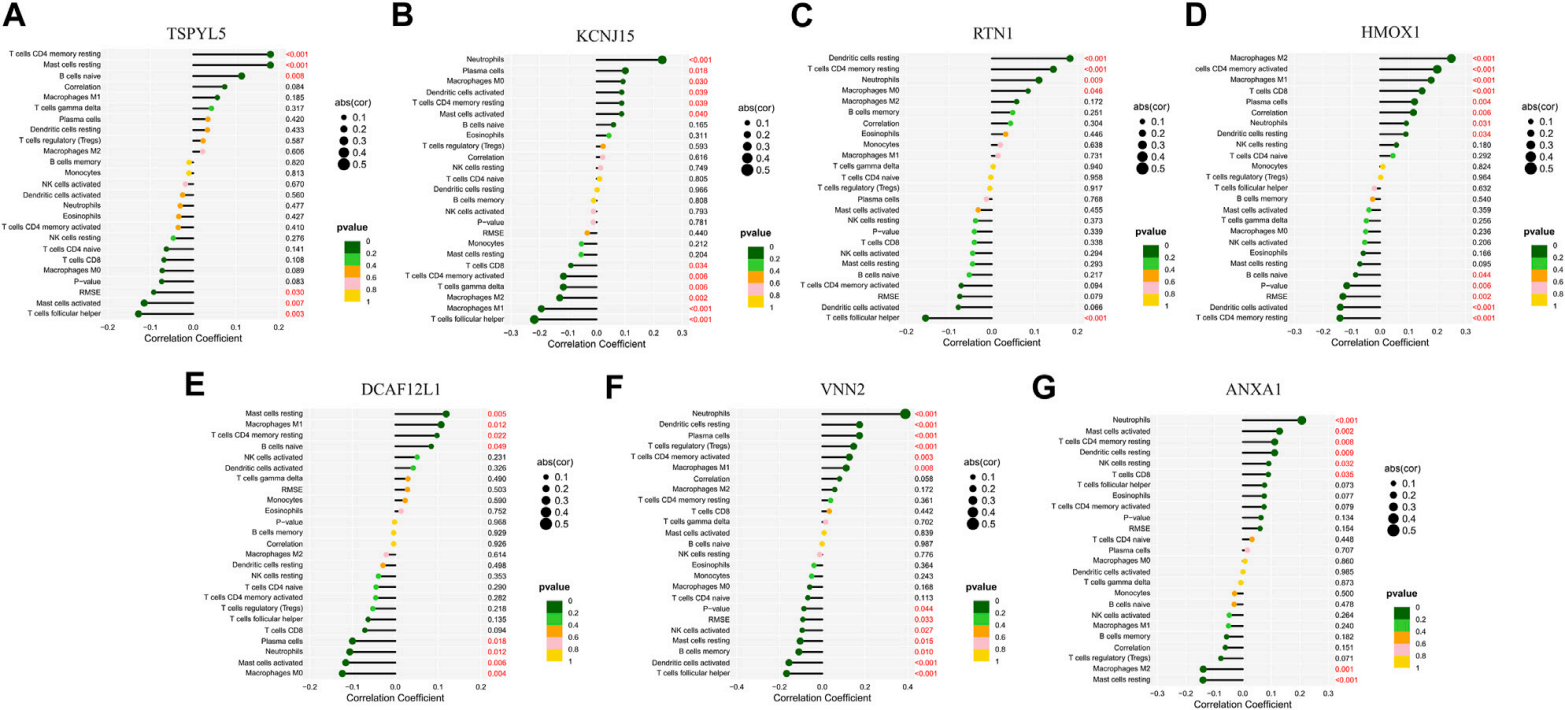
Lollipop chart of regulatory relationships between the seven prognostic UCEC genes and immune cells. The regulatory relationships between TSPYL5 **(A)**, KCNJ15 **(B)**, RTN1 **(C)**, HMOX1 **(D)**, DCAF12L1 **(E)**, VNN2 **(F)**, and ANXA1 **(G)** and immune cells.

The key prognostic genes TSPYL5, KCNJ15, RTN1, HMOX1, DCAF12L1, VNN2, and ANXA1 in UCEC were found to have regulatory relationships with immune cells such as T cells, B cells, NK cells, and others (Figures 12A–G). Among them, RTN1 was positively correlated with resting dendritic cells, resting CD4 memory T cells, neutrophils, and M0 macrophages, and negatively correlated with follicular helper T cells. (Figure 12C).

## 4 Discussion

UCEC, the fourth most prevalent female malignancy worldwide, is a common tumor in the female reproductive system (Cai et al., 2021). Its incidence is increasing globally, especially in developed regions (Makker et al., 2021; Banz-Jansen et al., 2022; Crosbie et al., 2022), which can be attributed to the rising prevalence of obesity (Rahib et al., 2014; Lauby-Secretan et al., 2016). UCEC has a low survival rate and poor prognosis (Brooks et al., 2019; Liu et al., 2021a), with a 5-year overall survival rate of 15%–17% for recurrent UCEC (Makker et al., 2021). However, early diagnosis and treatment of UCEC lead to better outcomes (Oaknin et al., 2022). Therefore, early diagnosis and treatment are crucial for UCEC. PCOS is a common disorder among women of reproductive age, with a global prevalence ranging from 6% to 20% (Joham et al., 2021; Kumariya et al., 2021). Obesity and low-grade inflammation associated with obesity are common features of PCOS (Barrea et al., 2021). Women with PCOS have an increased risk of developing tumors, including UCEC, ovarian cancer, breast cancer, and others (Gadducci et al., 2005; Xu et al., 2017; Meczekalski et al., 2020; Li Z. et al., 2022; Mitric and Bernardini, 2022). However, clinical outcomes of patients with the same tumor stage vary considerably, suggesting that conventional staging techniques may not accurately predict cancer prognosis (Xu et al., 2017). This emphasizes the importance of genetic diagnosis and therapy. Therefore, our focus is to explore the pathophysiological association between UCEC and PCOS and identify potential genetic biomarkers of UCEC.

Our study identified 1949 DEGs in UCEC, 2199 DEGs in PCOS, and 151 shared DEGs in UCEC and PCOS when compared to normal tissues. The shared DEGs PPI network of UCEC and PCOS was visualized using Cytoscape software, and Metascape-generated PPI networks were used for gene enrichment analysis (Li et al., 2019). GO and KEGG enrichment analysis of the 151 shared DEGs through the Metascape online database revealed that they were primarily enriched in leukocyte activation. Through model construction and internal verification using the TCGA database, TSPYL5, PARVG, KCNJ15, RTN1, CTSW, HMOX1, DCAF12L1, VNN2, and ANXA1 were identified as prognostic genes for UCEC within the shared DEGs of UCEC and PCOS. Additionally, the UCEC high-risk group exhibited a shorter survival time and worse prognosis compared to the low-risk group. External validation with additional datasets confirmed that TSPYL5, KCNJ15, RTN1, HMOX1, DCAF12L1, VNN2, and ANXA1 were key prognostic genes for UCEC. Analysis of these seven key prognostic genes using Gepia revealed that higher expression of RTN1 was associated with longer RFS in UCEC patients, indicating that detecting RTN1 expression may aid in the prognosis assessment of UCEC patients. Furthermore, RTN1 was identified as a potential marker in both UCEC and PCOS. Several studies have indicated that the seven key prognostic genes identified in this study for UCEC, including TSPYL5, VNN2, ANXA1, and DCAF12L1, are associated with tumor occurrence and development (Feng et al., 2020; Soler et al., 2020; Hernandez-Meza et al., 2021; Lu et al., 2021). In particular, DCAF12L1 has been considered a methylated gene contributing to the development of UCEC (Lu et al., 2021). Moreover, diabetes is a risk factor in both PCOS and UCEC (Lauby-Secretan et al., 2016; Patel, 2018; Liao et al., 2021), and HMOX1, RTN1, and KCNJ15 are considered diabetes-related genes (Okamoto et al., 2012; Meng et al., 2021; Meng et al., 2022).

Great progress has been made in the treatment of UCEC, but advanced or recurrent patients still present treatment challenges (Zhang Y. et al., 2022). Immunotherapy is a promising treatment approach that utilizes drugs to enhance the immune cells’ infiltration in the tumor microenvironment and activate the immune system to recognize and attack cancer cells (O'Donnell et al., 2019; Tao et al., 2021; Li X. et al., 2022; Marin-Jimenez et al., 2022; Zhang Y. et al., 2022). This method can effectively reduce the damage to healthy cells caused by treatment (Marin-Jimenez et al., 2022). Checkpoint inhibitor immunotherapy has revolutionized the treatment of various tumors, including UCEC (Marin-Jimenez et al., 2022). Pembrolizumab, a checkpoint inhibitor, has demonstrated efficacy in treating tumors with TMB, and pembrolizumab/lenvatinib has been used in the treatment of UCEC with high TMB (Marin-Jimenez et al., 2022). The results of this study on the immune infiltration of UCEC and PCOS-shared DEGs revealed differences in the expression of immune cells such as NK cells, CD8^+^ T cells, macrophages, and mast cells between patients with high and low TMB in UCEC. Further analysis of the seven key prognostic genes of UCEC indicated regulatory relationships with immune cells. However, the main limitation of this study is the lack of clinical validation experiments, as only samples from the TCGA database, GSE34526 dataset, GSE17025 dataset, and GSE48301 dataset were included. Although the altered expressions of TSPYL5, KCNJ15, RTN1, HMOX1, DCAF12L1, VNN2, and ANXA1 are associated with UCEC survival, TMB, immune infiltration, and immune cell regulation, it does not necessarily imply that the risk prediction model can be applied in actual clinical practice to provide a basis for immunotherapy.

In summary, this study successfully developed and validated a potential marker model for UCEC and PCOS, which identified TSPYL5, PARVG, KCNJ15, RTN1, CTSW, HMOX1, DCAF12L1, VNN2, and ANXA1 as prognostic genes for UCEC. Among these genes, TSPYL5, KCNJ15, RTN1, HMOX1, DCAF12L1, VNN2, and ANXA1 were found to be associated with the survival time, TMB, and immune infiltration in UCEC. RTN1 may serve as a novel immunotherapy biomarker for both UCEC and PCOS.

## Data Availability

The datasets GSE34526, GSE17025, and GSE48301 for this study can be found in the GEO database https://www.ncbi.nlm.nih.gov/geo/. The dataset UCEC for this study can be found in the TCGA database https://portal.gdc.cancer.gov/.
