# Supplementary figures and images for: Screening and validation of potential markers associated with uterine corpus endometrial carcinoma and polycystic ovary syndrome based on bioinformatics methods
Source: Front Mol Biosci. 2023 Jun 9;10:1192313. doi: 10.3389/fmolb.2023.1192313 (PMC10288877; doi:10.3389/fmolb.2023.1192313)

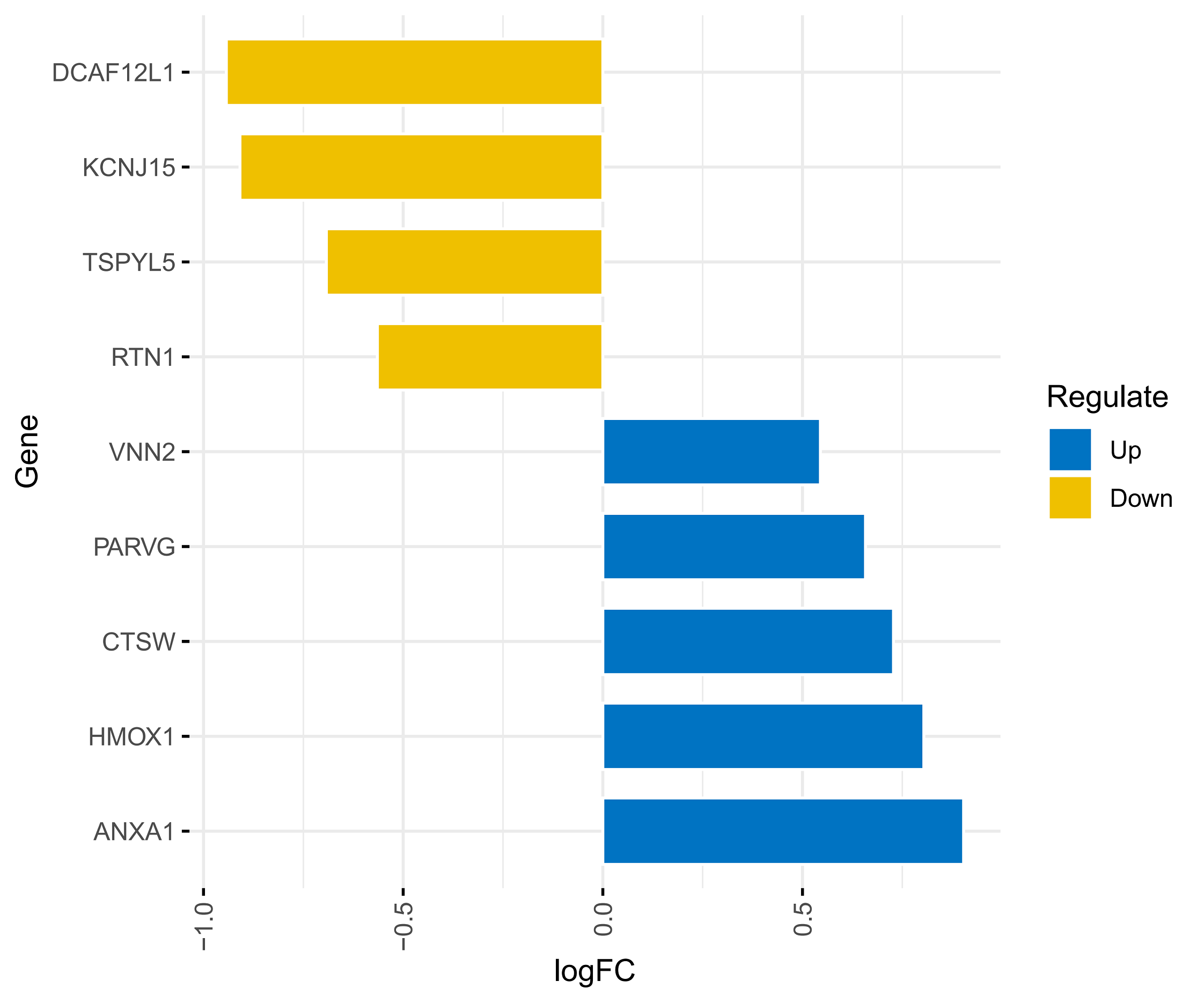

Supplement: Supplementary file 1 [file Image2.TIF]

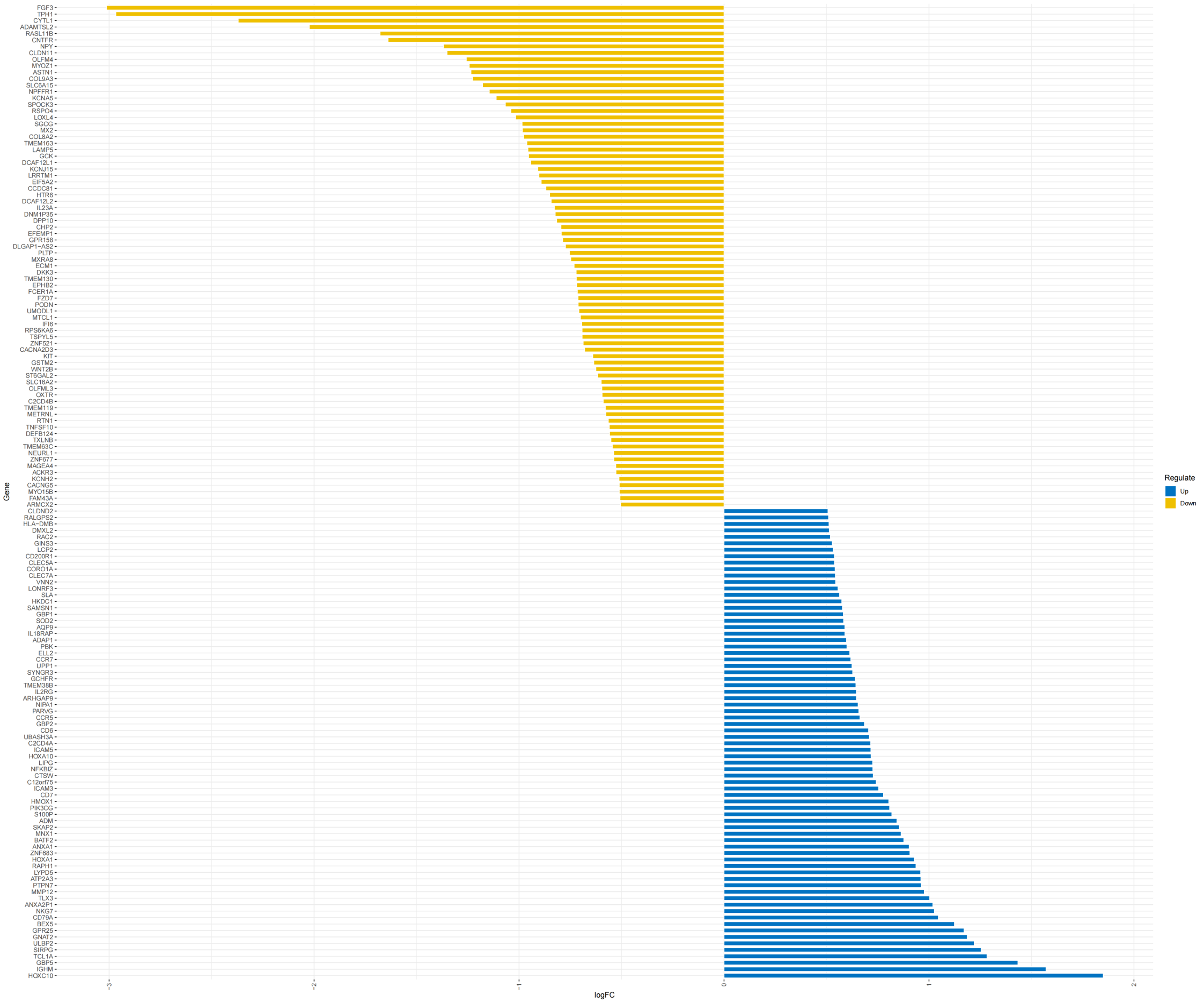

Supplement: Supplementary file 2 [file Image1.TIF]
